# Impact of intra-arrest fluid loading with different doses of crystalloid infusion on hemodynamics in experimental cardiac arrest

**DOI:** 10.1186/cc14500

**Published:** 2015-03-16

**Authors:** R Skulec, A Truhlar, R Parizkova, Z Turek, D Astapenko, J Dudakova, V Cerny

**Affiliations:** 1 Charles University in Prague, University Hospital Hradec Kralove, Czech Republic; 2 Emergency Medical Service of Central Bohemian Region, Kladno, Czech Republic; 3 J.E. Purkinje University, Masaryk Hospital, Usti nad Labem, Czech Republic

## Introduction

Fluid loading during cardiopulmonary resuscitation for nonhypovolemic cardiac arrest remains controversial. Thus, we conducted an experimental study comparing the impact of two different doses of balanced crystalloid infusion on hemodynamics in a porcine model of ventricular fibrillation.

## Methods

Ventricular fibrillation was induced for 15 minutes in 19 anesthetized domestic pigs. Before induction, the animals were randomized to receive either 1,000 ml (34 ± 3 ml/kg, group A, *n *= 7) or 500 ml (16 ± 2 ml/kg, group B, *n *= 7) of balanced crystalloid solution or to undergo no fluid loading during CPR (group C, *n *= 5). After spontaneous circulation (ROSC) was restored, the animals were observed for 90 minutes.

## Results

In all groups, significant increase of intracranial pressure followed by its decrease after ROSC was observed. While in groups B (from 12 ± 2 to 18 ± 2 mmHg, *P *< 0.05) and C (from 13 ± 1 to 18 ± 3 mmHg, *P *< 0.05) it was comparable (*P >*0.05), the rise of intracranial pressure in group A was significantly higher (from 12 ± 3 to 23 ± 3 mmHg, *P *< 0.05). Whereas coronary perfusion pressure was lower in group A than in control group C during volume loading, fluid infusion induced its mild increase in group B (group A: 12.1 ± 2.4, group B: 16.0 ± 2.6, group C: 13.6 ± 2.8 mmHg, *P *= 0.043). Decrease of cerebral perfusion pressure was equal in all groups. Cardiac index 10 minutes after ROSC significantly differed among all groups (group A: 8.9 ± 2.2, group B: 7.1 ± 1.3, group C: 4.9 ± 1.9 l/minute/m^2^, *P *= 0.007) and the dose of crystalloid infusion during cardiac arrest positively correlated with cardiac index increase (*r *= 0.815, *P *< 0.001).

## Conclusion

Fluid loading during CPR had significant impact on hemodynamics in our experimental model. While a high dose led to unintentional increase of intracranial pressure and decrease of coronary perfusion pressure, a low dose did not affect intracranial pressure and was associated with mild increase of coronary perfusion pressure during cardiac arrest.

